# The Neurobiology of Zika Virus: New Models, New Challenges

**DOI:** 10.3389/fnins.2021.654078

**Published:** 2021-03-29

**Authors:** Luciana Monteiro Moura, Vinicius Leati de Rossi Ferreira, Rafael Maffei Loureiro, Joselisa Péres Queiroz de Paiva, Rafaela Rosa-Ribeiro, Edson Amaro, Milena Botelho Pereira Soares, Birajara Soares Machado

**Affiliations:** ^1^Hospital Israelita Albert Einstein, São Paulo, Brazil; ^2^Gonçalo Moniz Institute, Oswaldo Cruz Foundation (IGM-FIOCRUZ), Bahia, Brazil; ^3^University Center SENAI CIMATEC, SENAI Institute of Innovation (ISI) in Advanced Health Systems (CIMATEC ISI SAS), National Service of Industrial Learning – SENAI, Bahia, Brazil

**Keywords:** Zika virus, cell death, neuroimaging, brain abnormalities, neurodevelopment, congenital Zika syndrome, artificial intelligence

## Abstract

The Zika virus (ZIKV) attracted attention due to one striking characteristic: the ability to cross the placental barrier and infect the fetus, possibly causing severe neurodevelopmental disruptions included in the Congenital Zika Syndrome (CZS). Few years after the epidemic, the CZS incidence has begun to decline. However, how ZIKV causes a diversity of outcomes is far from being understood. This is probably driven by a chain of complex events that relies on the interaction between ZIKV and environmental and physiological variables. In this review, we address open questions that might lead to an ill-defined diagnosis of CZS. This inaccuracy underestimates a large spectrum of apparent normocephalic cases that remain underdiagnosed, comprising several subtle brain abnormalities frequently masked by a normal head circumference. Therefore, new models using neuroimaging and artificial intelligence are needed to improve our understanding of the neurobiology of ZIKV and its true impact in neurodevelopment.

## Introduction

When the first neuroteratogenic effects of Zika virus (ZIKV) were described in 2015, microcephaly emerged possibly as a consequence of viral strain modifications and enhanced virulence. Recently, Congenital Zika Syndrome (CZS) cases are declining. However, ZIKV is evolving and spreading out, and thus the occurrence of a new epidemic should not be ruled out. Since first isolated in 1947 in Africa, several strains of ZIKV from African, Asian and American lineages were reported, and the continuous divergence and great diversification promote new interactions with vectors and hosts, impacting on its pathological properties, such as adaptation between human to human transmissions (Beaver et al., 2018). American lineage spread to Angola (Hill et al., 2019), and microcephalic cases were identified in Africa and Asia (WHO, 2019). By July 2019, 87 countries had reported evidence of autochthonous transmission, distributed across four of the six WHO Regions – Africa, the Americas, South-East Asia and the Western Pacific (PAHO/WHO, 2019; WHO, 2019). The first European vector-borne transmission of ZIKV was laboratory-confirmed in France (Giron et al., 2019). In addition to mosquito bite and prenatal maternal-fetal transmission, sexual transmission is of great concern (WHO, 2020), as well as the existence of persistent reservoirs with sustained high-level production of infectious ZIKV (Osuna et al., 2016; Dudley et al., 2019).

The mechanisms leading to the onset of severe abnormalities in fetuses exposed to the virus are still debated, involving many variables. CZS has a challenging framework, being characterized by a broad phenotypic spectrum of brain impairments (De Oliveira Melo et al., 2016) still poorly understood. Epidemiological reports are biased toward severe cases, presenting typical characteristics, such as microcephaly, not always present in the syndrome (França et al., 2016; Soares de Oliveira-Szejnfeld et al., 2016; Levine et al., 2017; Moore et al., 2017; UNDP, 2017; Cardoso et al., 2019). Importantly, limiting the analysis of brain impairments to the conspicuous radiological findings neglects many other subtle but still impacting alterations that are linked to ZIKV infection.

So far, rodent and *in vitro* models provided many insights in the investigation of ZIKV infection during embryonic and fetal neurodevelopment, but limitations are present. Stem cell-based *in vitro* models ideally address cell-type specificity (Ming et al., 2016), but they do not reproduce tissue and brain complexity faithfully (Rosenfeld et al., 2017), as well as how neurons and glial cells interact. *In vivo* rodent models can provide helpful details of the second trimester of brain development, but still lack complexity in the neurogenic cortical layers (Cadwell et al., 2019). In addition, rodent cells might differentially express receptors for ZIKV entry (Hastings et al., 2017). A complementary approach would be the use of brain-region specific organoids (e.g., forebrain organoids) (Qian et al., 2016). To investigate the late gestation and postnatal periods, non-human primate models are particularly important (Dudley et al., 2019); high costs and ethical concerns, however, are involved. Consequently, the application of advanced neuroimaging techniques associated with artificial intelligence (AI) can be valuable alternatives to improve our understanding of neurobiological underpinnings of ZIKV.

In this review, we address the most likely chain of events implicated in the atypical neurodevelopment of CZS. Consequently, we propose a shift in the diagnostic boundaries to include the great heterogeneity of subtle brain impairments (Waldorf et al., 2018a; Nielsen-Saines et al., 2019), which are difficult to trace with conventional neuroimaging approaches.

## ZIKV Infection and CZS Diagnosis are Modulated by Many Variables

**FIGURE 1:**
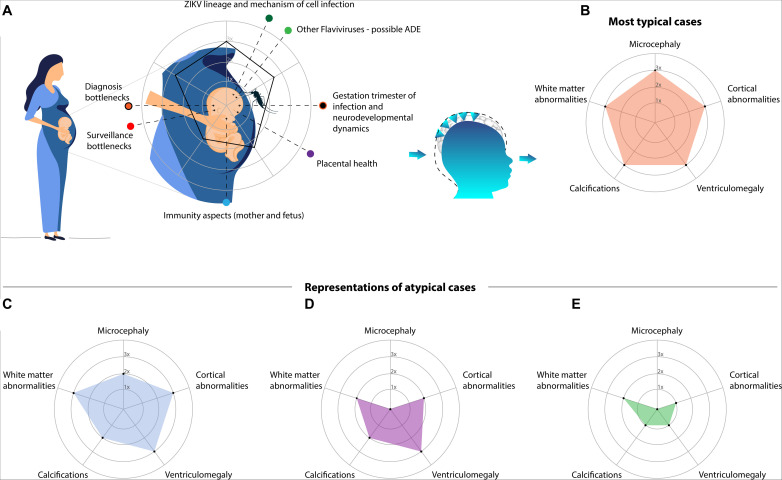
Although ZIKV is well established as the cause of the CZS, the spectrum of outcomes is highly variable. **(A)** Depicts a schematic diagram of prenatal exposure to multiple variables. In this period, time-varying events and covariates hamper the precision in predicting the outcome, for which artificial intelligence (AI) algorithms may be crucial. The most important event is the timeline of the infection: presumably the earlier is the infection, the worse are the impairments. However, many other variables and possible confounders are present, such as: the viral lineage and the mechanism of infection; overlapping infections of other flaviviruses that might lead to an antibody-dependent enhancement (ADE); misdiagnosis due to surveillance pitfalls or intrinsic diagnostic difficulties; immune-response of the mother (including placental health) and fetus. In addition, four examples of the CZS spectrum are represented in radar charts (axes with arbitrary values). Alterations driving the different phenotypes are grouped and oversimplified. **(B)** Is the pentagon, a typical and severe case which is readily detected, not necessarily diagnosed due to a possible lack of laboratorial confirmation. The other phenotypes are frequently neglected: **(C)** represents less severe cases (in comparison to the typical one), though noticeable, in the lack of laboratorial confirmation it might depend on the monitoring; **(D)** represents the pseudo-normocephalic cases, clinical alterations and neuroimaging may be crucial; **(E)** represents mild cases with subtle alterations within the gray and/or white matter, which are particularly challenging to detect and consequently the most neglected.

Although the causal association between ZIKV and CZS is clearly established, predicting the outcome after prenatal infection within the spectrum of the syndrome is challenging. This occurs due to two major factors: the lack of objective criteria for CZS and the intricate associations between many covariates and possible confounders that remain unknown (Figure 1A).

The lack of objective criteria refers to the fact that no diagnostic method alone can accurately define cases, since maternal symptoms are frequently mild, resulting in the loss of the viral detection window. Ultimately, diagnosis is based on other information, such as perinatal history, clinical-radiological findings, laboratory tests (such as blood analysis) (see Supplementary Material), used to boost the *diagnostic probability*. Therefore, subjects are frequently labeled in studies as highly probable, suspected, or unconfirmed cases (Victora et al., 2016; Adebanjo et al., 2017; Carvalho et al., 2019; Venturi et al., 2019). Often, only the temporal evolution course with cognitive and behavioral deficits will define the diagnosis.

**FIGURE 2:**
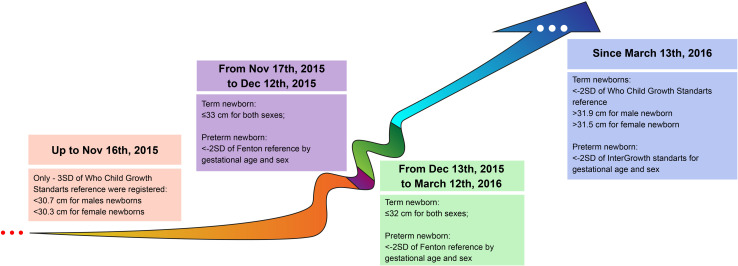
Evolution of diagnostic criterion based on head circumference (HC) between 2015 and 2016 in Brazil (the epicenter of cases). Before the first wave of cases, HC cutoff was more restrictive (<30.7 cm for males and <30.3 cm for females), from Nov 17th, 2015 when the epidemic was clearly established the cutoff was altered to ≤33 cm for both sexes in the attempt to track new cases. This lack of distinction between sexes was kept up to March 12th, 2016, which might have overestimated particularly female cases.

Although CZS cases became notorious due to microcephaly, this characteristic has many causes (Von der Hagen et al., 2014; Rosenfeld et al., 2017; Voekt et al., 2017; Alvarado-Socarras et al., 2018). Primary or prenatal microcephaly is usually a consequence of an abnormal development of the central nervous system mainly driven by the decreased number of neurons but not restricted to (Woods, 2004; Strafela et al., 2017). It corresponds to a smaller head circumference (<2SD, severe if <3SD) when compared to ethnically-, age-, and sex-matched controls (WHO, 2016). ZIKV infection of neural progenitor cells (NPC) can cause autophagy, apoptosis, and mitosis abnormalities (Souza et al., 2016) resulting in severe congenital malformations (Cugola et al., 2016). When caused by a secondary brain destruction (fetal brain disruption sequence), is accompanied by fetal skull collapse and redundant folded skin (de Souza A. S. et al., 2018). Usually, microcephaly is adopted as inclusion criterion to CZS (Victora et al., 2016) and its definition has dramatically changed over time (Figure 2; Box 1 for details). However, using head circumference as the main diagnostic cutoff for CZS could mask more subtle, but still important, abnormalities (van den Pol et al., 2017; Waldorf et al., 2018b, a; Figueiredo et al., 2019; Rosa-Fernandes et al., 2019; Walker et al., 2019). Therefore, diagnosis should be based on the identification and measurement of features describing the multidimensional nature of the phenotypic spectrum (Figures 1B–E).

**BOX 1. Time-Varying Definition of Microcephaly.**

Until November 16, 2015, only newborns with three standard deviations (SD) below the mean HC were considered microcephalic (but not mild cases between −2SD and −3SD). Currently, these cases are referred to as severe microcephaly. Until December 13, 2016, similar cut-off values were adopted for both sexes (WHO, 2007; Victora et al., 2016; Brasil, 2020). Among the 29 countries reporting ZIKV-related microcephaly (and other central nervous system malformations), Brazil has the highest number of cases, with only a few coming from Asia, Africa, Oceania and other countries of the Americas (WHO, 2017, 2019). This epidemiological profile is still under the scrutiny of the scientific community. The most investigated hypotheses related to this endemic characteristic might be related to characteristics common to Flaviviruses and viral mutation in general, since all lineages of ZIKV are neurotropic (Rosenfeld et al., 2017).

The bottlenecks in surveillance.

Misdiagnosis of ZIKV maternal infection impacts surveillance and child diagnosis. Infection peaked in 2016, when 651,590 cases were reported in the Americas, of which 199,614 were clinically confirmed. Between 2016 and 2018, clinically or laboratory-confirmed cases decreased by about 98% (*n* = 3,598), and increased 75% in 2019 (*n* = 6,287) (PAHO/WHO, 2019). Data collection and epidemiological conclusions are challenging due to revisions in diagnostic clinical criteria (Cuevas et al., 2016), along with limitations of laboratory testing and frequently asymptomatic or mild manifestations of the disease (Centers for Disease Control and Prevention, 2015; Musso and Gubler, 2016; Flamand et al., 2017; WHO, 2018) easily confounded with other arboviruses, such as dengue (Cardoso et al., 2015). Low-income countries are subject to many limitations of laboratory tests availability: in 2018, only 6 and 11% of cases were laboratory-confirmed in Brazil and in the Americas, respectively (WHO, 2019; Brasil, 2020). ZIKV tests are subject to serological cross-reaction with other flaviviruses (Musso and Gubler, 2016; Ribeiro G. S. et al., 2017) and the acute phase has a short detection window for laboratory testing (e.g., 7 days for blood serum samples) (Sharp et al., 2019), which is quite limiting to track the infection in real time. Immunoglobulin binding tests, even plaque-reduction neutralization tests (PRNT), recommended by the CDC to distinguish ZIKV from DENV, are affected by cross-reactive antibodies (Musso and Gubler, 2016; Lindsey et al., 2018; Sharp et al., 2019). Frequently, CZS cases labeled as probable are only investigated postnatally based on assumptions about maternal/fetal infection and medical records, in such cases neuroimaging is a crucial tool to support diagnosis.

The associations between many covariates and possible confounders remain unknown, combining all the puzzling events that define the trajectory of the infection. Similarly, epidemiological-based studies and clinical pharmacological trials face this problem, defined as exposure-affected time-varying confounding that demands robust paradigms of investigation, for which AI approaches should be more suitable (Clare et al., 2019). Graph-based models would be especially helpful to integrate non-imaging information in supervised and non-supervised approaches of machine learning, which have been successfully used in convolutional neural networks to predict complex disorders such as autism spectrum disorder and Alzheimer (Parisot et al., 2018).

In the CZS investigation AI algorithms should deal with high complexity paradigms involving multiple sources of information (Figure 1A). It is expected that the earlier is the ZIKV infection during prenatal neurodevelopment the worse are the congenital abnormalities in fetuses, because a protracted viremia worsens the symptoms (Martinot et al., 2018; Lima G. P. et al., 2019). Therefore, gestation trimester plays a pivotal role in the outcomes due to the intrinsic dynamics of brain development. Apparently, infection in pediatric populations result in mild complications but biases must be considered (Ramond et al., 2020), such as the fact that most studies devoted attention to the prenatal exposure. The early postnatal ZIKV infection may be crucial to the neurodevelopment (Mavigner et al., 2018), but it remains largely unknown in humans, particularly regarding the subtle alterations within the gray and white matter. The temporal precision of infection timeline is crucial but on the other hand diagnostic imprecisions boosted by surveillance bottlenecks must also be considered. In this context, the short window of ZIKV detection, cross-reactive antibodies and bottlenecks in the surveillance add extra layers of complexity (for more details Box 1); as well as the time-varying immunity aspects and ZIKV lineage (Souza et al., 2016) and the pivotal role of the placental health (Szaba et al., 2018; Barbeito-Andrés et al., 2020) (for more details Box 2).

**BOX 2. The pivotal role of the placental health.**

In early gestation, a pro-inflammatory process mediated by the placental dendritic cells is critical to promote the successful implantation of embryos (Dekel et al., 2010). In the successive trimesters there is a shift to an anti-inflammatory environment (Robinson et al., 2018) and, consequently, the placental barrier becomes less vulnerable to the virus. The innate immune system mediates this process through interferons (IFNs) that decisively antagonize viral infection in the host cells (Grant et al., 2016). When primitive placental trophoblasts differentiate, they become more resistant to ZIKV through the expression of IFN-stimulated genes (Corry et al., 2017). IFNs perform a wide range of activities, such as adaptive immune responses, but they can also contribute to the pathogenesis of diseases (Davidson et al., 2015). Placental damage and fetal miscarriage can be triggered by type I IFNs through apoptosis signaling – presumably IFN signaling mediates fetal pathology rather than the viral load (Yockey et al., 2018). The placental health is actually a better prognostic of adverse fetal outcome than the level of ZIKV RNA (Szaba et al., 2018).

Although ZIKV-specific IgG antibodies show a protective immune effect, including heterologous viral genotype strains in the maternal and fetal tissue (Turner et al., 2017), they have a variable neutralizing potency (Ojha et al., 2018). This likely implies a role for other variables, such as ADE, which enhances the ability of ZIKV to cross the placental barrier (Brown et al., 2019; Langerak et al., 2019), a reaction that is mediated by neonatal Fc (FcRN) receptors (Rathore et al., 2019). The role for these receptors is to prolong the half-life of IgG by mediating the transplacental transport from mother to fetus, providing newborns with humoral immunity (Stapleton et al., 2015).

Co-circulating flaviviruses is another possibly important source of confusion that enhances ZIKV infection through the so-called antibody-dependent enhancement (ADE), that might occur when a secondary infection enhances viremia (Diamond et al., 2019). Phylogenetically related viruses have very similar envelope proteins, which are the primary targets of the neutralizing antibody response (Bardina et al., 2017). ADE occurs when maternal non-neutralizing heterologous antibodies (from the primary flavivirus serotype, other than ZIKV) bind to ZIKV, increasing infection severity (Hermanns et al., 2018; Rathore et al., 2019). In addition, it was demonstrated that microcephaly severity is correlated with DENV-specific antibodies in mouse fetuses whose mothers were treated with these antibodies (Rathore et al., 2019). However, findings are still contradictory, as some studies argue in favor of a protective role DENV cross-infection (Bardina et al., 2017; Brown et al., 2019; Ngono and Shresta, 2019; Rodriguez-Barraquer et al., 2019). Because most of studies are restricted to *in vitro* and animal models (Rosenfeld et al., 2017; Martinot et al., 2018; Dudley et al., 2019; Langerak et al., 2019), human epidemiological-based investigations would be crucial to this understanding (Rathore et al., 2019).

## The Aftermath of ZIKV Infection: Prenatal Neuronal Impairments

**FIGURE 3:**
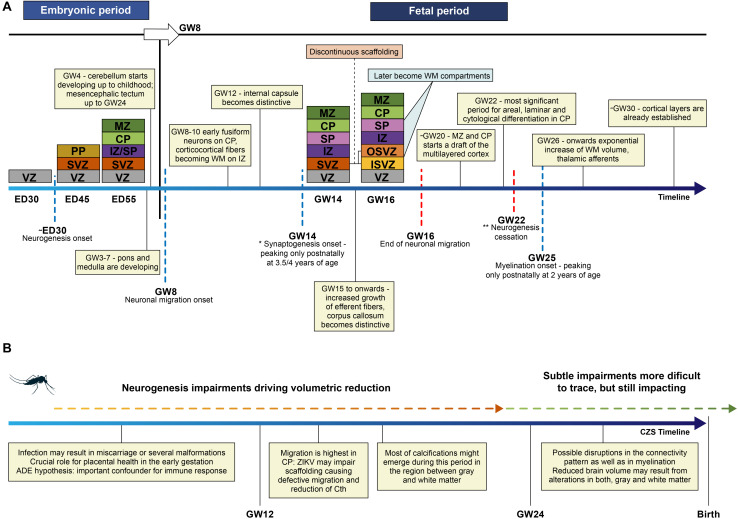
**(A)** Schematic representation of the embryonic layers in the typical developing neuroepithelium is depicted in colors, proportional intervals in the timeline were not respected. ED – embryonic days; GW – gestation week; Cth – cortical thickness; VZ – ventricular zone, PP – preplate, SVZ – subventricular zone, IZ – intermediate zone, SP – subplate, CP – cortical plate, MZ – marginal zone, OSVZ – outer subventricular zone, ISVZ – inner subventricular zone. Discontinuous scaffolding is a singular pattern of migration that splits SVZ into two discontinuous layers; OSVZ is supposed to drive the tangential or radial expansion of the human brain through the outer radial glia cells. *Considering the maturation of the membranes; ** referring to the neurogenesis waves in the whole brain, though some regions, i.e., hippocampus keeps active processes of neurogenesis in the adult life. **(B)** Some key events that may occur are represented in the timeline of prenatal neurodevelopment after ZIKV infection. These events depend not only on the period of the infection but also on the many covariates. Therefore, the severity of findings in CZS is highly variable.

Neurodevelopment during the embryonic and fetal period depends on the accurate development of several neural milestones (Marin-Padilla, 1970; Hasegawa et al., 1992; Bayer et al., 1993; Rakic and Zecevic, 2000; Howdeshell, 2002; Bystron et al., 2008; Deoni et al., 2016; Cadwell et al., 2019; Vasung et al., 2019; Figure 3A) and other surrounding variables. A *continuum* of fine-tuned and temporally orchestrated events must occur: neural tube formation, cell proliferation, migration, neuronal differentiation, key patterning of progenitor cells, and cell maturation (Van Ooyen, 2011). These events depend on different factors: the gestation stage, gene expression under epigenetic mechanisms of control, the exposure to maternal and/or environmental factors, and several interactions in the molecular and cellular levels (Van Ooyen, 2011; Gapp et al., 2014; Szaba et al., 2018; Cadwell et al., 2019). Neurogenesis begins around the 30th embryonic day, and declines in the middle of the second trimester (Bystron et al., 2008; Vasung et al., 2019). From the early embryonic days until birth (and extending to the postnatal period), dynamic and intricate processes involving transient cells and layers are pivotal to the typical patterning of the brain, by the way they proliferate, migrate and finally organize themselves (Bystron et al., 2008). Therefore, insults during this period, such as ZIKV infection, are prone to be incorporated in connectivity patterns (Andersen, 2003).

It is well characterized that ZIKV is able to cross the placental barrier through the transmembrane protein AXL, then the fetal blood-brain barrier, replicating in different organs and systems (Richard et al., 2017; Li et al., 2018). In fibroblasts and endothelial cells, AXL receptors sustain viruses attached to the cell, being key to promote invasion (Hamel et al., 2015; Liu et al., 2016). However, once reaching the embryo, the exact mechanism of how ZIKV infects human NPC remains only partially understood. It has been suggested that its invasion relies on a more complex interaction of AXL receptor, since its experimental ablation does not prevent the viral invasion (Wells et al., 2016). Indeed, ZIKV would be able to infect different types of neuronal cells through the exploitation of multiple cell surface receptors and cytosolic components (Esteves et al., 2017). This impairs multiple cellular processes, causing cell death, inflammatory response, disrupting the normal biological development (Rosa-Fernandes et al., 2019).

Most studies have focused on investigating these processes on stem cells and NPC, given the remarkable impact of these cells in the neurodevelopment. Particularly, cells located in the ventricular and subventricular zone and cortical plate (CP) (Figure 3A) are susceptible to ZIKV infection (Zhang W. et al., 2019), causing disruption of crucial events of early embryonic neurodevelopment (Garcez et al., 2016). The most susceptible period for ZIKV infection is the first and second trimesters, causing disruptions in the initial pool of NPC in the following steps of differentiation (Qian et al., 2016; Waldorf et al., 2018a; Rosa-Fernandes et al., 2019; Zhang W. et al., 2019). Neurogenesis is disrupted in the prenatal setting (van den Pol et al., 2017), although the exact mechanism of cell death that could be enrolled in this disruption is still a matter of debate (Ojha et al., 2018).

Radial glial proliferation is downregulated during the infection during ZIKV infection (Coolen et al., 2012; Homem et al., 2015; Zhang H. et al., 2019). At the beginning of the second trimester of gestation, when migration is highest at the CP, ZIKV was found in ventricular surface cells, extending to the radial progenitor glial cells, impairing the linear scaffold for the migration of neuronal precursors (Rosenfeld et al., 2017; Figure 3B). Cell to cell interaction is pivotal in the correct neuronal migration: it has been shown that ZIKV promotes the differential expression of genes likely enrolled in the defective migration (Aguiar et al., 2020). This impairment is compatible with the histological report of gray matter heterotopia (De Oliveira Melo et al., 2016) and supports the high prevalence of epilepsy in children affected by ZIKV after birth (Van Der Linden et al., 2018). The defective neuronal migration can also be responsible for agyria or lissencephaly, pachygyria, and polymicrogyria, the last two being reported most frequently (Soares de Oliveira-Szejnfeld et al., 2016; de Souza A. S. et al., 2018), and can also cause a reduction of cortical thickness, resulting in smaller brains (Homem et al., 2015).

### Neural Cell Death

Cell death is essential to orchestrate normal neurogenesis. Apoptosis is a modulatory event crucial to morphogenesis that helps to sculpt brain development (Yamaguchi and Miura, 2015). Programed cell death peaks for neuron precursors in the first trimester of gestation, and for glial cells in mid-gestation (Rakic and Zecevic, 2000). Embryonic apoptosis likely influences the actual size of the neuronal population. In turn, fetal apoptosis is hypothesized to crucially shape axonal connectivity alongside differentiation and synaptogenesis (Rakic and Zecevic, 2000). Impairments of the coordinated cell death may cause consequences to the typical development of the brain structure that are not fully understood. ZIKV cell infection induces a cascade of events that can result in different mechanisms of cell death. Shortly, after bypassing cell defenses through endocytosis, non-structural proteins (NPs) are synthesized starting from ZIKV RNA, using the cell elements and organelles to raise new virions (Ojha et al., 2018). In turn, their subunits mislead and control the immune defense through interference in cell processes to facilitate viral replication (Li et al., 2016; Lima G. P. et al., 2019) (for more details Box 3).

**BOX 3. ZIKV replication within neural cells.**

Viral replication depends on the impairment of different types of pro-inflammatory IFNs signaling, along with other cell mechanisms whose kinetics is dependent on the viral lineage (Beaver et al., 2018; Ojha et al., 2019). For instance, ZIKV^*BR*^ diminishes the response of I IFNs stimulated genes, leading to an enhanced viral burden and resulting in a chronic inflammatory process, which is likely associated with microcephaly (Lima M. C. et al., 2019). NS5 ZIKV non-structural protein promotes degradation of the interferon effector STAT2 (a transcriptional activator) and consequently results in I IFN degradation, impairing viral block replication (Grant et al., 2016; Li et al., 2016). IFN signaling depends on the interactions with entry cell receptors, such as AXL (a cell surface TAM-family receptor) (Lemke, 2013; Meertens et al., 2017). AXL is pivotal for flaviviruses and is expressed in several cells, such as some placental cells, astrocytes, microglia, oligodendrocytes, human radial glia and NPCs (Li et al., 2016; Akkermann et al., 2017; Esteves et al., 2017; Meertens et al., 2017). Its precise role remains uncertain; it could act as an entry factor for ZIKV endocytosis (Meertens et al., 2017; Ojha et al., 2019) or antagonize type I IFN signaling (Chen et al., 2018). It is recognized that AXL is more efficiently used by ZIKV than by DENV, disrupting mechanisms mediated by the arrest-specific protein 6 (Gas6) to protect cells from stress-induced apoptosis (Esteves et al., 2017). Toll-like receptors, another class of receptors, have also been shown to be involved in ZIKV replication and inflammatory responses, inducing autophagy in microglia and astrocytes (Ojha et al., 2019).

Apoptosis pathway requires cleaved-caspase-3 at the final stage of activation, typically ending in a silent cell death and disassembly (Nuñez et al., 1998). These events were replicated by many studies using human and rodent NPC, in neurons and glia using 2D and neurosphere cell cultures infected by ZIKV (Cugola et al., 2016; Souza et al., 2016; Tang et al., 2016; Zhang et al., 2017; Lima M. C. et al., 2019; Rosa-Fernandes et al., 2019). Low concentrations of betulinic acid showed to be neuroprotective, increasing the number of SOX2 + NPC in ZIKV-infected brain organoids (Cavalcante et al., 2020). Although apoptosis is able to reduce the neuronal cell populations affecting the brain structure, other types of cell death are involved, such as pyroptosis (He et al., 2020). The presence of inflammatory cytokines and necrotic tissue suggests the occurrence of pyroptosis characterized by the activation of inflammasomes, caspase-1 and gasdermin D, being strongly inflammatory and differing from apoptosis, which is a silent process. This occurs due to the leakage of intracellular content and cleavage of IL-1β and IL-18, two pro-inflammatory cytokines (Shi et al., 2015; de Sousa J. R. et al., 2018). The analysis of brain tissue collected from newborn fatal cases of ZIKV-linked microcephaly revealed increased levels of inflammasome activation of IL-1β in parenchyma suggesting a pivotal role of pyroptosis (de Sousa J. R. et al., 2018) and, so far, pyroptosis is confirmed in NPC (He et al., 2020).

Pathogenic cell death may explain two major features of CZS: parenchymal reduction and intracranial calcifications, likely correlated (Pool et al., 2019). Cell perturbations of Ca^2+^ homeostasis impair physiological processes and the development of morphological characteristics (i.e., neurites) (Orrenius et al., 2003; Van Ooyen, 2011), and mediate many intracellular processes related to cell death (Orrenius et al., 2003). Calcium deposits are related to iron accumulation, a process that is possibly preceded by oxidative stress and chronic inflammation mediated by microglia in ZIKV infection (Snyder-Keller et al., 2020). However, the exact mechanism through which intracranial calcifications are generated is unknown. Disruptions of homeostatic cell death are able to cause reduction in the number of cells and to trigger inflammatory processes interfering in the typical neurodevelopment during the prenatal period.

Experimental findings are still puzzling, and the whole picture is fragmented due to the intricate characteristics of cell processes, embryonic/fetal neurodevelopment. Although the main effect of ZIKV occurs in the embryonic period, brain impairments are persistent and, importantly, they might be continuous extending to the postnatal period. Indeed, the brain is still vulnerable to significant structural and functional complications at later developmental stages including ZIKV, and even postnatally (Mavigner et al., 2018; Carvalho et al., 2019; Dudley et al., 2019; Raper et al., 2020).

## The Aftermath of ZIKV Infection: Postnatal Investigation

### Distinctive Brain Abnormalities

Neuroradiological findings are routinely used to support CZS diagnosis, pre and postnatally. However, they become crucial together with the clinical records considering the following scenario: a pregnant woman exposed to ZIKV with no clinical manifestation of the infection and also no evident brain abnormalities in the fetus that could attract attention during prenatal exams. In such cases, the neuroradiological findings described in the CSZ literature (Table 1; Mehrjardi et al., 2017; Ribeiro B. G. et al., 2017; Sanz Cortes et al., 2018; de Souza A. S. et al., 2018) should be decisive to provide the diagnosis.

**TABLE 1:** The neuroradiological findings frequently described in the CZS literature.

**Neuroimaging findings**	**Details**
Microcephaly	Head circumference below 2 standard deviations below the mean for the gestational age and sex, or below the 3rd percentile. It may be absent in some cases
Intracranial calcifications	More common in the cortical-subcortical junction, secondarily in the basal ganglia, and/or the thalamus
Ventriculomegaly	Ventricular septations or synechia can be found
Parenchymal volume loss	Associated with enlarged supratentorial subarachnoid space
Malformations of cortical development	Lissencephaly, pachygyria, simplified gyral pattern, polymicrogyria, schizencephaly, and gray matter heterotopia
	Polymicrogyria and pachygyria are predominantly found in the frontal lobes
Corpus callosum abnormalities	Agenesis, hypogenesis, and dysgenesis
Delayed myelination and dysmyelination	Wallerian degeneration and/or development arresting of: pontocerebellar connections, corticobulbar and corticospinal tracts. Degeneration of the long descending tracts in the brain stem and spinal cord
Cerebellar and brain stem abnormalities	Hypoplasia is the most frequent abnormality
Collapsed skull with overlapping sutures	Associated with exuberant occipital protuberance and redundant scalp skin
Other abnormalities	Enlarged cisterna magna

However, it is well known that the detection of biomarkers remains a challenge even in the presence of clear lesions (Batalle et al., 2018), particularly for CZS that might be confounded with other TORCH (the acronym for T. gondii, rubella virus, cytomegalovirus, herpes simplex virus, and many others) (Vigliani and Bakardjiev, 2014). This group of congenital infections largely overlaps CZS. Therefore, we highlight the intracranial calcifications pattern of distribution as the most promising marker to perform this distinction (Soares de Oliveira-Szejnfeld et al., 2016; Levine et al., 2017) and to provide insights from prenatal neurodevelopmental events. Pattern recognition analysis would be key to deepen our understanding (Shen et al., 2017).

Intracranial calcifications tend to be larger and denser in the gray-white matter transition in ZIKV-infected fetuses, but are also found in the basal ganglia, thalamus, brain stem, cerebellum, and cortical regions (Soares de Oliveira-Szejnfeld et al., 2016; Levine et al., 2017). The presence of intracranial calcifications in gray-white matter transition may be partially explained by the infection of cells localized in the subplate (SP) (Figure 3B), a key region of cortico-cortical connectivity with a critical role in the vulnerability of the pre and perinatal brain (Kostović et al., 2014). SP has a pivotal role as an axonal compartment (Vasung et al., 2019) and in programed cell death (Rakic and Zecevic, 2000), which is disrupted in CZS. However, they are not always present and, in some cases such as in prenatal brain (perhaps mirroring embryonic and fetal stages of neurodevelopment), they assume a distribution that tends to be periventricular, similar to cytomegalovirus (CMV) infection (Soares de Oliveira-Szejnfeld et al., 2016; Levine et al., 2017). Therefore, a combination of features would be crucial to support CZS diagnosis, including to the maximum informative inputs: brain imaging, and if available, data from clinical and laboratorial exams. By pooling all of these many variables and their possible associations, machine learning enhances the power of generalization and, thus, would classify cases with greater accuracy (Bzdok et al., 2018), going beyond linear models to include non-linear combinations between features.

The neuroimaging spectrum of CZS likely comprises a combination of highly correlated features, each one in a different level of severity, emerging as many different manifestations of the syndrome (Figures 1B–E). Typical and severe cases attracted most of the attention devoted to CZS. Normally they are readily detected in prenatal and/or postnatal screening (Figure 1B). Pseudo-normocephalic cases, in which the absence of microcephaly is masked by a severe ventriculomegaly, or even by enlargement of supratentorial subarachnoid space (Aragao et al., 2016) will make diagnosis more challenging (Figure 1D). Therefore, the diagnosis is likely to rely on the progression of the neurological impairments. At least, the most difficult cases to identify are those presenting with subtle abnormalities (Figure 1E). They might pass unnoticed until the first manifestations of impairments in late childhood. Importantly, such alterations are not accessed by the radiological imaging of routine.

In this circumstance, machine learning would be particularly robust, making possible the identification, classification and quantification of patterns (Shen et al., 2017). Identification and selection of features using supervised learning (Chandrashekar and Sahin, 2014) or detection of new emerging patterns and hidden features would be performed using unsupervised learning, particularly deep learning (DL) (Goodfellow et al., 2016). DL is also fundamental in processing data through computer vision (Esteva et al., 2019), that is, it is crucial in the search of patterns in neuroimages. To circumvent the demand of large datasets, multicentric collaboration and/or analysis of retrospective real-world datasets from public services is suggested.

### Subtle and Neglected Brain Abnormalities

#### Gray Matter

**FIGURE 4:**
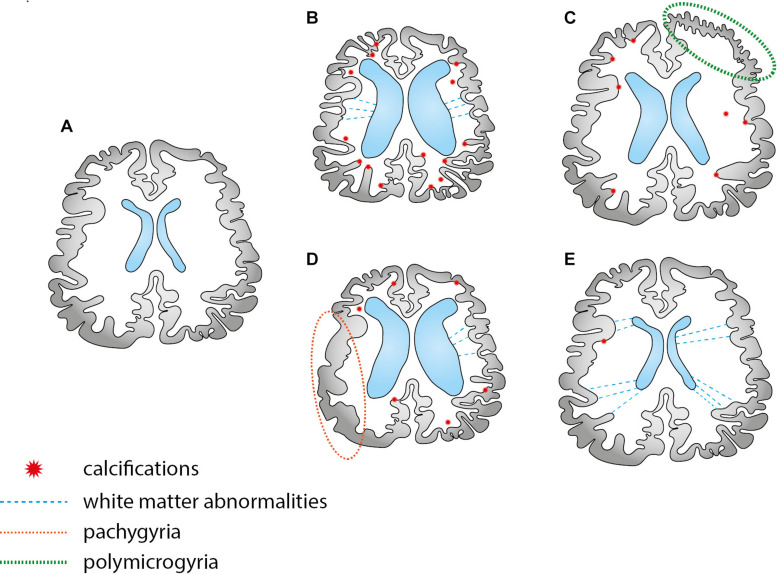
A few schematic examples of possible combinations of brain abnormalities in the CZS. There are two types of abnormalities: those that can be easily identified by the traditional imaging scans, such as calcifications and ventriculomegaly in CT scans; subtle alterations that are difficult to trace, requiring specific MRI sequences (frequently investigated in the scope of research). Scheme **(A,B)** depicts two typical images, normal developing and microcephalic brain, respectively. Schemes **(C,D)** depicts migration cortical abnormalities, such as pachygyria and polymicrogyria, better investigated by specific structural MRI sequences. Case **(E)** depicts a normocephalic case with subtle abnormalities in the white matter, better investigated by diffusion MRI techniques.

Severe cases of CZS have attracted most of attention, especially those cases running with microcephaly, ventriculomegaly and intracranial calcifications. Here, we argue that limiting the syndrome to the reduced head circumference and radiological findings may underestimate subtle but impacting neuroimaging alterations (Figure 4). These alterations underlie the dimensional diagnostic approach and demand specific strategies of imaging that go beyond conventional neuroradiological findings. Therefore, we provide an overview of the neuroimaging literature to investigate the subtle alterations of CZS, focusing on *in vivo* MRI of the postnatal brain for research purposes. Some considerations about medical imaging advantages and limitations are available in Table 2 (Pugash et al., 2008; Conner et al., 2014; Batalle et al., 2018; Makropoulos et al., 2018; Vasung et al., 2019).

**TABLE 2:** Brain imaging for clinical purposes: the main advantages and limitations.

		**Advantages**	**Limitations**	**Observations**
**Prenatal**	Obstetric US	Cost-effective Radiation-free High availability, especially for remote places and low-income regions Best method for microcephaly and brain calcification screening	Less sensitive than MRI in the detection of parenchymal abnormalities Operator-dependent	Difficult to visualize the entire brain and subarachnoid space (Pugash et al., 2008) To frame diagnosis other means should be used (Conner et al., 2014) Lacks correspondence with MRI (Batalle et al., 2018)
	Fetal MRI	Better depiction of brain parenchymal abnormalities (e.g., cortical malformations, brain stem, and cerebellar hypoplasia) T2-weighted imaging is recommended. But T1-weighted provides more precise information about cell density and myelination, and can detect calcification	High cost Less sensitive than US in detecting discrete/dispersed calcification Low availability, especially in the public health services in developing countries Artifacts due to standing wave and conductivity effects, acoustic noise, fetal movement, and maternal breathing	*Ex vivo* fetal brain MRI undergoes a rich technical improvement allowing for the investigation of early events (Vasung et al., 2019)
**Postnatal**	Transfontanellar US	The above-mentioned for obstetric US	The above-mentioned for obstetric US Depends on the fontanelle opening	
	Head CT	Gold standard for calcification depiction Better spatial resolution, allowing for 3-dimensional skull reconstructions Shorter acquisition time than MRI, rarely requiring patient sedation	Ionizing radiation Low contrast resolution	
	Head MRI	Radiation-free Higher contrast resolution Although less sensitive than CT, MRI can detect intracranial calcification with great accuracy through specific sequences Gold standard for detection of parenchymal abnormalities, malformation of cortical development, delayed myelination, or dysmyelination, and posterior fossa abnormalities	High cost Low availability, especially in public health systems in developing countries Longer acquisition time, which may require patient sedation Highly sensitive to motion, being especially difficult to implement protocols with toddlers	

Head circumference may be a predictor of successful rehabilitation in children within the severe spectrum of CZS (Carvalho et al., 2019), but it does not reveal the underlying causes of the parenchymal loss. Structural MRI provides valuable anatomic information and volumetric quantification: up to 3 months of age, T2 weighted sequences are recommended to avoid neonatal biases, one of them being myelination, that impairs reliable conclusions about cortical changes (Di Donato et al., 2017). After that, T1 contrast allows the accurate calculation of cortical components and also the evaluation of defective neuronal patterns of migration that ultimately drives findings such as pachygyria and polymicrogyria (Figures 4C,D). Volumetric alteration encodes two different aspects: cortical thickness and surface area, and their measurements approximate the underpinnings of the cortical architecture (Ecker et al., 2015). According to the radial-unit hypothesis (Rakic, 1995), cortical thickness is related to the intermediate progenitor cells, which exclusively produce neurons and later migrate radially to arrange the ontogenetic columns as radial units, thus, amplifying the number of cells in the column. In turn, surface area is related to the radial unit progenitor cells, which increase the number of columns by the tangential multiplication in the apical ventricular surface. The outer subventricular zone (OSVZ) is supposed to drive this expansion (Cadwell et al., 2019). This could bring more interpretability to ZIKV repercussions in the gray matter, especially related to the small foci of heterotopias. We underscore that volume reduction is intuitively associated with neuronal loss, but many other underlying processes can be implicated, such as myelination (Oliveira et al., 2010), which is considered to be a key confounder of cortical thickness until 24 months of age (Di Donato et al., 2017).

Beyond cell death and volumetric reductions, findings point out that infected cells can stay alive but morphologically affected by neurite disruption (Goodfellow et al., 2018). Mature neurons show extensive downregulation of synapse-related proteins and decreased synapse density (Rosa-Fernandes et al., 2019). This is also compatible with the differential expression of cell adhesion components that might disrupt neurite outgrowth and axon guidance in CZS (Aguiar et al., 2020). Impairments in the pattern of neurons and glia were also described (Waldorf et al., 2018a). Mild cases without any other clear lesion may be particularly affected by these alterations, which are difficult to trace. Hippocampal neurogenesis, a phenomenon which is still a matter of debate (Lucassen et al., 2020) would be disrupted by ZIKV leading to severe impairments in neurocognitive and psychological behaviors (Waldorf et al., 2018b), since robust infections were found in CA1 and CA3 (van den Pol et al., 2017; Figueiredo et al., 2019).

#### White Matter

WM abnormalities can vary from the mesoscale, detected in the investigation of tracts, such as the malformations reported in the corpus callosum (Table 1), to the microstructural scale, such as neurite impairments (Rosa-Fernandes et al., 2019). Frequently, WM and cortical alterations are coupled (Figure 4D). However, if isolated and occurring within the microstructure, they are more difficult to track. Although enhanced apoptosis has been reported in neurons in CZS, glial cells are also an important source of volume reduction, including oligodendrocytes and astrocytes at late stages of neurodevelopment (Li et al., 2018). Still according to this study using mouse models, it is suggested a switch of ZIKV infection to the impairment of gliogenesis, particularly oligodendrogenesis. Astrocytes play a fundamental role in blood-brain barrier maintenance (Haddad-Tóvolli et al., 2017). They are specially infected in the first gestation trimester, and widespread foci of infection are consistently found throughout the brain (coupled with vascular injury), impacting myelination and neurotransmitter modulation (van den Pol et al., 2017). ZIKV disrupts mitochondrial processes in astrocytes leading to oxidative stress, DNA damage, and cell death that would be an important source of motor neurons apoptosis (Ledur et al., 2020). Consequently, reactive gliosis is induced and has been described in the frontal lobes of CZS cases of death shortly after birth. To better investigate gliosis it would be beneficial using specific MRI sequences in association with positron emission tomography (PET) (Cavaliere et al., 2020). Noteworthy, neuronal infection secondarily would surpass the glial infection, supporting the hypothesis of axonal transport between long-distance regions in the brain (van den Pol et al., 2017).

Particular attention should be devoted to investigate the white matter (WM) integrity more deeply with specifically designed MRI sequences (e.g., diffusion-weighted imaging), given several reports of disruption involving cell surface TAM-family receptors in a variety of WM cells infected by ZIKV (Waldorf et al., 2016; Meertens et al., 2017; Li et al., 2018; Mavigner et al., 2018; Ojha et al., 2019; Ledur et al., 2020). These receptors have a pivotal role in ZIKV infection and also in myelination (Akkermann et al., 2017). We highlight that the impact of ZIKV infection in the WM may be critical during early childhood, given its pivotal role in brain plasticity underlying the consolidation of functional connectivity (Deoni et al., 2016).

Studies of structural connectivity through tract reconstruction (tractography), combined with respective alterations in the cortical areas, should be useful to evaluate prognosis in moderate and severe cases of CZS. For instance, frequently reported impairments in CZS can be found in the motor domain, in brainstem abnormalities, as well as in degeneration of the long descending tracts in the brain stem and spinal cord (De Oliveira Melo et al., 2016; Mlakar et al., 2016; Chimelli et al., 2017; de Souza A. S. et al., 2018). Myelination disruption in the hemispheric white matter, internal capsule and cerebellum, as well as the absence of descending fibers result in the underdevelopment of pontocerebellar, pyramidal, and corticospinal tracts (Chimelli et al., 2017). Transdiagnostic traces, such as those also present in other pathologies, should improve our insights about CZS and guide future studies to selected tracts and regions of interest. Functional connectivity investigation is unlikely in severe cases due to sedation, but mild cases would benefit from this approach.

For investigating all these abnormalities, a profusion of cutting-edge MRI sequences is available. Nevertheless, sophisticated MRI sequences frequently require prolonged acquisition periods, which overburden patients and make images more prone to movement artifacts, and consequently demands more of preprocessing and brain segmentation strategies, especially in fetal and neonatal samples (Makropoulos et al., 2018). Fortunately, alternatives are currently available: such as adapted sequences for clinical scanners, to investigate the brain microstructure of neonates (Kunz et al., 2014), joint sequences (multi-modal approach) to address microstructural and specific properties of the tissues (Cercignani and Bouyagoub, 2018), AI algorithms to allow neuroimaging post-processing strategies (Moeskops et al., 2016).

## Conclusion

Environmental and intrinsic physiological variables have a complex interplay in determining the trajectory of ZIKV infection. Although *in vitro* and animal models provided so far invaluable information, the literature still lacks a clear understanding of the CZS. The comprehension of the different trajectories and its associated outcomes is challenging and requires robust paradigms of investigation. AI would allow not only the inclusion of multiple cofactors but it would be crucial in processing brain images. Precisely, it might be necessary to reconsider the epidemic definition for CZS in light of the spectrum of brain impairments, especially of mild cases. The spectrum of pseudo normality may be larger than previously considered, limited by many intrinsic diagnostic and surveillance inaccuracies. It includes subtle, but still significant brain alterations (Waldorf et al., 2018b) that cannot be assessed by conventional neuroimaging. Decisions must be taken to tailor the best intervention strategy at the earliest opportunity. However, misdiagnoses impair these decisions, particularly during optimal therapeutic windows in the early childhood aiming at stimulating brain plasticity. Long-term monitoring of normocephalic children exposed *in utero* to ZIKV has already been suggested to trace neurocognitive deficits and the enhanced risk of early onset of neurocognitive and psychiatric conditions (Kapogiannis et al., 2017; Waldorf et al., 2018a,b; Nielsen-Saines et al., 2019). The data reviewed in this manuscript support this suggestion and emphasize the need of longitudinal cohorts and of increased surveillance in health services and schools, targeting the mild and unnoticed cases. Monitoring in the school setting is particularly important since the first wave of children possibly affected by ZIKV is only now entering the preschool age. Neurological deficits might emerge and be framed as learning deficits.

## Author Contributions

LM conceived and designed the idea. LM, VF, RL, RR-R, and MS wrote and reviewed the manuscript content and text. JP, EA, and BM reviewed the manuscript content and text. All authors contributed to the article and approved the submitted version.

## Conflict of Interest

The authors declare that the research was conducted in the absence of any commercial or financial relationships that could be construed as a potential conflict of interest.
