# Supplementary material for: The Neurobiology of Zika Virus: New Models, New Challenges
Source: Front Neurosci. 2021 Mar 29;15:654078. doi: 10.3389/fnins.2021.654078 (PMC8059436; doi:10.3389/fnins.2021.654078)
Supplement: Supplementary file 1 [file Table_1.DOCX]

**SUPPLEMENTARY MATERIAL**

**Definitions of confirmed cases of CZS in Brazil** (Brasil 2015; Brasil 2017)

**Period: 2015 to 2016**

Cases in Brazil between 2015 and 2016 were classified in accordance with the “Vigilance protocol and response to the occurrence of microcephaly and/or alterations of central nervous system” (Brazil, 2015), published on March 24^th^, 2016. When necessary, cases identified before this date were reclassified. In this protocol, **confirmed cases** followed the criteria described below:

*GROUP 1: Identification of fetuses with alterations in the Central Nervous System (CNS) during gestation*

Criteria for confirmed cases, according to specific exams:

- Case confirmed following clinical-radiological criterion:
  - Confirmed case suggestive of congenital infection through clinical- radiological criterion: notified cases that were not excluded by the criteria that follow below.
- Case confirmed following laboratory criterion:
  - Confirmed case suggestive of congenital STORCH infection: notified cases that present with specific laboratorial result for syphilis, toxoplasmosis, rubella, cytomegalovirus, or herpes simplex. Analysis of blood or urine samples OR amniotic fluid, if recommended by clinical protocols.
  - Confirmed case suggestive of congenital infection of ZIKV: notified cases that present with conclusive result for ZIKV. Analysis of blood or urine samples OR amniotic fluid, if recommended by clinical protocols.

*GROUP 2: Identification of miscarriages suggestive of congenital infection*

According to specific exams:

- Confirmed case:
  - Confirmed case suggestive of congenital STORCH infection: notified cases that present with specific laboratorial result for syphilis, toxoplasmosis, rubella, cytomegalovirus or herpes simplex. Analysis of blood or urine samples OR miscarriage sample, if recommended by clinical protocols.
  - Confirmed case suggestive of congenital infection of ZIKV: notified cases that present with conclusive result for ZIKV. Analysis of blood or urine samples OR miscarriage sample, if available.

*GROUP 3: Identification of stillbirths and elective terminations suggestive of congenital infection*

- Confirmed case:
  - Confirmed case suggestive of congenital STORCH infection: notified cases that present with specific laboratorial result for syphilis, toxoplasmosis, rubella, cytomegalovirus or herpes simplex. Analyzed from blood or urine samples of the expectant or puerperal mother OR tissue of the stillborn.
  - Confirmed case suggestive of congenital infection of ZIKV: notified cases that present with conclusive result for ZIKV. Analyzed from blood or urine samples of the expectant or puerperal mother OR tissue of the stillborn.
- Probable case:
  - Probable case of microcephaly suggestive of congenital infection: notified case, whose mother had presented with exanthematous rash during gestation, when a laboratory investigation is not possible.

*GROUP 4: Identification of neonates with microcephaly*

Classification for microcephaly:

- Neonate born before 37 weeks of gestation, presenting with head circumference (HC) < 2 SD for gestational age and sex, according to the INTERGROWTH - 23th table.
- Neonate born after 37 weeks of gestation, presenting with HC < to 31.5 centimeters for females and 31.9 for males, equivalent to < 2SD for neonate age and sex, according to the WHO table.

Confirmed cases according to specific exams:

- Confirmed case using imaging exams (ultrasound, computed tomography or magnetic resonance)
  - Confirmed case of neonate with microcephaly suggestive of congenital infection: notified case presenting with alterations suggestive of congenital infection, using any imaging method, not presenting laboratorial results (if the lab results are received after classification, the case should be reclassified).
- Confirmed case using laboratorial criteria:
  - Confirmed case suggestive of congenital STORCH infection: notified cases with microcephaly AND that present conclusive laboratorial result for syphilis, toxoplasmosis, rubella, cytomegalovirus or herpes simplex. Analyzed from neonate and/or maternal samples.
  - Confirmed case suggestive of ZIKV infection: notified cases with microcephaly AND that present specific and conclusive laboratorial result for ZIKV. Analyzed from neonate and/or maternal samples.

**Period: From 2017**

On December 12^th^, 2016, a preliminary version of the document entitled, “Integrated guidelines of surveillance and attention to healthcare within the scope of Public Health Emergencies of National Importance” was published (Brazil, 2017). From the first epidemiological week of 2017 forward, new definitions of cases were adopted. Below are the definitions of confirmed cases:

- Confirmed case of congenital infection without etiological definition:
  - Notified case of a newborn, child, fetus, or stillborn with results of imaging exams that describe two or more signs and symptoms (in the imaging or clinical exam) presented in Annex A, **WITH** reporting of an exanthem or fever without a defined cause during gestation AND without lab results that point to STORCH+Zika, either due to a mistake when collecting the mother’s or neonate’s lab sample; **OR** with a negative or inconclusive lab result for STORCH+Zika, analyzed from a sample from the mother or neonate, following all the specific guidelines for each disease and testing availability.
- Confirmed case of congenital infection by the Zika virus:
  - Suspected case with signs and symptoms (from imaging or clinical exams) systematized in Annex A, under the following conditions:
    - Neonate: Positive or reagent result for the Zika virus in the best-performing test, with the neonate’s sample, if the quality requirements for the test are met (sample collected up to the 8^th^ day of life, type of material, processing, etc.) **AND** a negative or inconclusive result in at least 1 STORCH (syphilis, toxoplasmosis, rubella, CMV, or HSV) in a sample from the neonate or mother (during gestation), **AND** two or more signs and symptoms (from imaging or clinical exams) presented in Annex A.
    - Fetus: From a pregnant woman with a reagent/positive result for the Zika virus in the best-performing test, if the quality requirements for the test are met (time of sample collection, type of material, processing, etc.) **AND** a negative or inconclusive result in at least 1 STORCH (syphilis, toxoplasmosis, rubella, CMV, or HSV) in a sample from the pregnant mother, **AND** with an imaging exam showing two or more alterations, following Annex A.
- Confirmed case of congenital infection from STORCH:
  - Suspected case with signs and symptoms (from imaging or clinical exams) systematized in Annex A, preferably for cases notified within the first 48 hours of life, under the following conditions:
    - Neonate: Positive or reagent result for at least 1 of the STORCH (syphilis, toxoplasmosis, rubella, CMV or HSV) from a sample of the neonate or the mother (during gestation) in the best-performing test, if the quality requirements for carrying out the test are met (time of sample collection, type of material, processing, etc.), **AND** a negative or inconclusive result for Zika from the neonate sample, **AND** two or more of the signs and symptoms presented in Annex A.
    - Fetus: From a pregnant woman with a positive or reagent result for at least one of the STORCH (syphilis, toxoplasmosis, rubella, CMV or HSV) from a sample of the pregnant woman in the best-performing test, if the quality requirements for the test are met (time of collection, type of material, processing, etc.) **AND** a negative or inconclusive result for Zika from the sample from the pregnant woman, **AND** with an imaging exam showing two or more alterations, following Annex A.
- Confirmed case of congenital infection from STORCH+Zika (coinfection):
  - Suspected case with the signs and symptoms (from imaging or clinical exams) systematized in Annex A, preferably for cases notified within the first 48 hours of life, under the following conditions:
    - Neonate: Positive or reagent result for at least 1 of the STORCH (syphilis, toxoplasmosis, rubella, CMV or HSV) from a sample of the neonate or the mother (during gestation) in the best-performing test, if the quality requirements for carrying out the test are met (time of sample collection, type of material, processing, etc.), **AND** a positive or reagent result for Zika from a sample from the neonate, **AND** two or more of the signs and symptoms (from imaging or clinical exams) presented in Annex A.
    - Fetus: From a pregnant woman with a positive result or reagent for at least 1 of the STORCH (syphilis, toxoplasmosis, rubella, CMV or HSV) in a sample of the pregnant woman in the test with the best performance, if the quality requirements for the test are met (time of collection , type of material, processing etc.) And positive result or reagent for Zika in a sample of the pregnant woman AND with an image exam showing two or more changes, following Annex A.
    - Fetus: From a pregnant woman with a positive or reagent result for at least 1 of the STORCH (syphilis, toxoplasmosis, rubella, CMV or HSV) from a sample of the pregnant woman in the best-performing test, if the quality requirements for the test are met (time of sample collection, type of material, processing, etc.), **AND** a positive or reagent result for Zika from a sample from the pregnant woman, **AND** with an imaging exam showing two or more alterations, following Annex A.
- Confirmed death (miscarriage, fetal death, stillbirth, or death after birth) from congenital infection from Zika virus:
  - Stillbirth or death after birth: Positive or reagent result for the Zika virus in a sample from the stillbirth or neonate in the best-performing, if the quality requirements for the test are met (time of sample collection, type of material, processing, etc.) **AND** a negative or inconclusive result for at least one STORCH (syphilis, toxoplasmosis, rubella, CMV or HSV) from a sample of the mother, stillborn or neonate.
  - Miscarriage or fetal death: Pregnant woman with a positive or reagent result for the Zika virus in the best-performing, if the quality requirements for carrying out the test are met (time of sample collection, type of material, processing etc.), from samples collected during pregnancy, **AND** a negative or inconclusive result for at least one STORCH (syphilis, toxoplasmosis, rubella, CMV or HSV) from the pregnant woman’s sample.
- Confirmed death (miscarriage, fetal death, stillbirth, or death after birth) from congenital infection from STORCH:
  - Stillbirth or death after birth: Positive or reagent result for at least one STORCH (syphilis, toxoplasmosis, rubella, CMV or HSV) from a sample from the mother, stillborn, or neonate in the best-performing test, if the quality requirements for the test are met (time of sample collection, type of material, processing, etc.) AND a negative or inconclusive result for Zika from the stillborn or neonate sample.
  - Miscarriage or fetal death: Pregnant woman with positive or reagent result for at least one STORCH (syphilis, toxoplasmosis, rubella, CMV or HSV) in the best-performing test, if the quality requirements for the test are met (time of sample collection, type material, processing, etc.), from samples collected during pregnancy, **AND** a negative or inconclusive result for Zika from the pregnant woman’s sample.
- Confirmed death (miscarriage, fetal death, stillbirth, or death after birth) from congenital infection from STORCH+Zika (coinfection):
  - Stillbirth or death after birth: Positive or reagent result for the Zika virus from a sample of the stillbirth or neonate in the best-performing test, if the quality requirements for the test are met (time of sample collection, type of material, processing, etc.) **AND** a positive or reagent result for at least 1 STORCH (syphilis, toxoplasmosis, rubella, CMV or HSV) from a sample from the mother, stillborn or neonate.
  - Miscarriage or fetal death: Pregnant woman with a positive or reagent result for the Zika virus in the best-performing test, if the quality requirements for the test are met (time of sample collection, type of material, processing etc.), from samples collected during pregnancy, **AND** a positive or reagent result for at least one STORCH (syphilis, toxoplasmosis, rubella, CMV or HSV) in a sample from the pregnant woman.

**Annex A – Main findings related to the congenital syndrome associated to Zika virus infection**

**Most common alterations identified during the pre-natal period**

| **Neurological alterations in imaging exams** | | **Facial dysmorphisms** | **Musculoskeletal** | **Other** |
| --- | --- | --- | --- | --- |
| Microcephaly (following guidelines of the reference tables)  Microencephaly  Posterior fossa alterations: cerebellar vermis dimorphisms  Ventriculomegaly (light, moderate, and severe – *ex vacuum*)  Hydrocephaly  Cerebral calcifications – disseminated  Synechiae  Dysgenesis of the corpus callosum  Schizencephaly / porencephaly  Tapering of the cortex  Prominent occipital | Craniofacial disproportion  Flat face  Microphthalmia  Retrognathia  Hypertelorism  Redundant skin of the scalp (*cutis rugata* and/or *cutis gyrata*) | | Compensatory hand and foot positioning (arthrogriposis proxy) | Amniotic fluid volume alteration (polyhydramnios) |

**REFERENCES**

Brasil M da S. 2015. Protocol for surveillance and response to the occurrence of microcephaly and / or central nervous system (CNS) alterations. Available from: www.saude.gov.br/svs

Brasil M da S. 2017. Integrated guidelines for surveillance and health care in the context of the Public Health Emergency of National Concern (PHEIC): procedures for monitoring changes in growth and development from gestation to early childhood, related to Zika virus infectio. Available from: http://portalarquivos.saude.gov.br/images/pdf/2016/dezembro/12/orientacoes-integradas-vigilancia-atencao.pdf
